# The influence of clinical and sociodemographic variables on employment and education for Danish youth with a psychotic disorder: a population-based national registry study

**DOI:** 10.1007/s00127-026-03085-5

**Published:** 2026-04-01

**Authors:** Trine Gade Bonnesen, Anne Petrea Hansen, Vivian Langagergaard, Mathilde Louise Søndergaard Graham, Sanne Jørgensen, Pernille Pedersen

**Affiliations:** 1 Department of Clinical Social Medicine and Rehabilitation, Gødstrup Hospital, Herning, Denmark; 2 DEFACTUM, Central Denmark Region, Evald Krogs Gade 16A, Aarhus, 8000 Denmark; 3 Department of Public Health, Aarhus University, Aarhus, Denmark

**Keywords:** Psychotic disorder, Employment, Education, Sociodemographic factors, Clinical factors

## Abstract

**Purpose:**

This population-based cohort study aimed to examine how clinical and sociodemographic characteristics are associated with employment and educational status 3 and 5 years after a first-episode psychotic disorder.

**Methods:**

The study included individuals diagnosed with a psychotic disorder between 2012 and 2017 in the Central Denmark Region. Register-based data were obtained from the CROSS-TRACKS cohort. Logistic regression analyses were used to determine the association between sociodemographic and clinical factors and the likelihood of being employed or enrolled in education at 3- and 5-year follow-up.

**Results:**

A total of 2,493 individuals aged 18–35 years were included in the analyses. At 3- and 5- years post-diagnosis, 19.6% and 21.6%, respectively, were employed or enrolled in education. In adjusted analyses, baseline employment or educational participation and being married were associated with higher odds of attachment at both follow-up time points. Female sex was associated with lower odds of attachment at the 3-year follow-up only, while schizophrenia was associated with lower odds of employment or educational engagement at the 5-year follow-up. More frequent psychiatric outpatient visits during the first two years post-diagnosis were associated with higher odds of attachment at both follow-up points, whereas psychiatric hospitalization was associated with lower odds.

**Conclusion:**

Marital status, employment or educational participation at diagnosis, and engagement with psychiatric outpatient care were associated with later attachment to employment or education, highlighting the importance of early and sustained support following first-episode psychosis.

## Introduction

Psychotic disorders, including schizophrenia, schizoaffective disorders, and brief psychotic disorders are among the most severe mental health conditions, with profound implications for both individuals and society [1–3]. In the global population, the lifetime prevalence of experiencing at least one psychotic symptom is approximately 12.5%, and only about 1% are diagnosed with schizophrenia [4]. These disorders typically emerge in late adolescence or early adulthood [5–7], and their impact is wide-ranging, affecting employment, education, social relationships, and the ability to live independently [3, 8, 9].

It has previously been shown that recovery rates following a first-episode of psychosis remain stable at approximately 38% over a 20-year period when recovery is defined using a combination of clinical and functioning criteria and that only approximately 14% of individuals with schizophrenia achieve such recovery [10, 11]. Employment plays a vital role in the recovery process by fostering social inclusion, reducing psychiatric hospitalizations, and providing a sense of purpose [12]. However, employment rates among individuals with first-episode psychosis are low – ranging from 10 to 20% during the first five years after diagnosis to 32.5% after eight years of follow-up [10, 13–15]. Regional data further underscore these challenges. In Denmark, 87% of individuals diagnosed with schizophrenia by the age of 25 were unemployed [16], while in Sweden, 80% relied on disability pension [17]. Psychotic disorders are also strongly associated with educational dropout and social isolation [18].

Employment outcomes are influenced by a range of sociodemographic and clinical factors. For instance, being married or previously married is associated with higher employment likelihood, while unemployment at discharge or readmission to the hospital within three years is linked to poorer outcomes [19]. Additionally, older age at diagnosis may predict better functioning [18], and factors such as stronger premorbid functioning, higher cognitive ability, and fewer concurrent symptoms are also associated with improved employment status [20].

Although the association between clinical and sociodemographic characteristics and functioning outcomes in psychotic disorders is well established, important gaps remain. In particular, there is limited evidence on how different types and intensities of healthcare utilization in the early post-diagnostic phase are associated with later attachment to employment or education. Existing research has largely focused on symptom remission or clinical outcomes, often without examining functioning outcomes such as employment and educational participation, which are central to long-term recovery. While psychosocial interventions have been shown to support social and occupational functioning in early psychosis, comparisons across studies are challenged by heterogeneity in outcome definitions and limited use of population-based data [21–23]. To address these gaps, the present population-based study aimed to examine how clinical and sociodemographic characteristics, including patterns of healthcare utilization, are associated with attachment to employment or education at three and five years following a first-episode psychotic disorder. By using comprehensive register data, the study provides both short- and longer-term assessments of functioning outcomes in a large, real-world cohort.

## Methods

### Study design and population

This retrospective population-based cohort study was conducted using data from the CROSS-TRACKS cohort [24]. From this cohort, we identified all individuals aged 18 to 35 years residing in the Central Region of Denmark with a first-episode of a psychotic disorder (ICD-10 codes F20-F29) between January 1st, 2012, and December 31st, 2017. A first-episode psychotic disorder was defined as the first-ever recorded diagnosis of a psychotic disorder in the Danish National Patient Registry; individuals with any prior registered diagnosis of a psychotic disorder before the index date were excluded. Follow-up continued until death or the end of the study period, allowing for a maximum follow-up of five years. Accordingly, the end of the study period was December 2022. The study population included individuals who were alive and eligible for labor market or educational participation. Therefore, individuals receiving disability pension at baseline (i.e. at the time of psychotic diagnosis) were excluded.

### Data sources

The Danish CROSS-TRACKS cohort integrates data from primary and secondary healthcare systems, municipal services, and national registries for individuals aged 18 and older in the Central Region of Denmark [24]. These data provide detailed information on diagnoses, treatment, socioeconomic factors, and public welfare enabling comprehensive analyses of health and social outcomes.

Study individuals were identified through the Central Population Registry (CPR) and linked across multiple data sources using their unique personal identification number. The Danish Register for Evaluation of Marginalization (DREAM) provided weekly information on receipt of social benefits [25], while the CPR supplied demographic data, including information on sex, age, and civil status [26]. Data from the primary health care system were obtained via public healthcare insurance records and municipal healthcare service registries [27]. The Danish National Patient Registry provided diagnostic information based on ICD-10 and included dates of admission and discharge [28], [29].

### Outcomes

Employment status was obtained from the DREAM database [25, 30], containing periods during which individuals received social benefits such as state educational grants, unemployment benefits, sickness absence benefits, or disability pension.

Employment was defined as periods with recorded labor market contribution or receiving benefits related to flexible job schemes. Educational enrollment was defined as periods during which individuals received a state educational grant, provided to all students enrolled in an education program. Employment and education were combined into a single outcome category and assessed at three and five years after the initial psychotic disorder diagnosis. All remaining individuals were classified as receiving public welfare benefits.

The validity of DREAM data has been validated through comparison with workplace-registered sick leave and self-reported income data [25], [30].

### Exposure variables

Civil status was categorized as unmarried, married or in a registered partnership, and divorced or widowed. Baseline employment status was categorized in the same way as the outcome variable. ICD-10 diagnoses were categorized as either schizophrenia (F20) or other psychotic disorders, including schizotypal disorder (F21), delusional disorder (F22), acute and transient psychotic disorders (F23), induced delusional disorder (F24), schizoaffective disorders (F25), other nonorganic psychotic disorders (F28), and unspecified nonorganic psychosis (F29). The number of contacts in the primary healthcare system including visits to general practitioners, physiotherapists, psychologists, and practicing psychiatrists was aggregated over the first two years following diagnosis and categorized as 0–20, 21–50, or more than 50 contacts.

The number of somatic and psychiatric hospitalization days and outpatient visits was recorded for the two years following diagnosis. These contacts were classified as somatic or psychiatric based on the department type and the primary diagnosis registered for each contact. Somatic hospitalization days were categorized as none or ≥ 1 day. Psychiatric hospitalization days were categorized as none, 1–30 days, or > 30 days.

Outpatient visits related to somatic and psychotic disorders were identified and categorized separately for the same two-year period.

### Statistical analyses

Characteristics of individuals at baseline and during the period 0–2 years thereafter were described using frequencies and percentages.

Logistic regression analyses were conducted to examine the association between sociodemographic and clinical factors and the likelihood of being employed or enrolled in education at three and five years following a first-episode of a psychotic disorder. Analyses were performed using crude and adjusted models, with adjustments for sociodemographic factors (sex, age, civil status, and baseline employment status) and clinical variables (diagnosis, number of hospitalization days, outpatient visits, and primary healthcare visits).

Analyses at the 3-year and 5-year follow-up were conducted among individuals alive at the respective time points. Individuals who died before a given follow-up were not included in the analyses at that time point, as they were no longer eligible for the outcome. Due to data limitations in the DREAM database, which only contains records up to November 2022, some individuals lacked complete data at the 5-year follow-up. These individuals were excluded from the corresponding analysis, as outcome status could not be determined at that fixed time point.

All estimates are reported with 95% confidence interval (CI) and statistical significance was set at *p* < 0.05. The statistical analyses were carried out using State/MP version 18.0 (StataCorp LLC).

## Results

### Study population

**Fig. 1:**
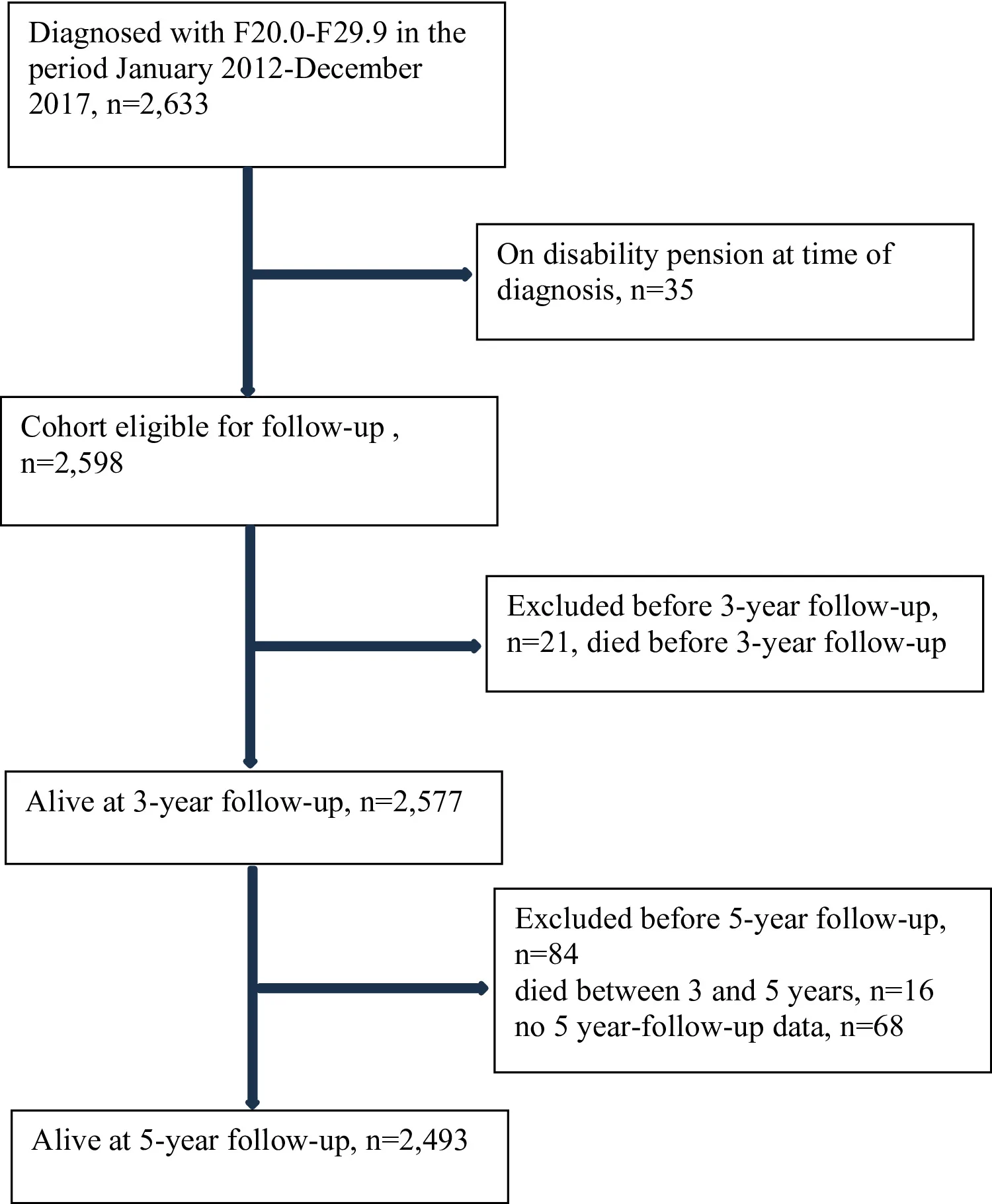
Flow chart of the study population

A total of 2,633 individuals were identified with a first-episode of a psychotic disorder. At baseline, 35 individuals were excluded due to receiving disability pension (Fig. 1). At the 3-year follow-up, 21 individuals were additionally excluded due to death, leaving 2,577 individuals eligible for analyses. At the 5-year follow-up, an additional 84 individuals were excluded due to death (*n* = 16) or missing follow-up data (*n* = 68), leaving 2,493 individuals for analyses (Fig. 1).

Table 1 presents sociodemographic and clinical characteristics at the time of the first recorded diagnosis of a psychotic disorder and during the first two years following diagnosis. The majority of individuals were male, and in the younger age group, were unmarried, and were not employed or enrolled in education at baseline (Table 1). Approximately 60% were diagnosed with other psychotic disorders, while the remaining individuals were diagnosed with schizophrenia. More than half of the population had fewer than 20 contacts with primary healthcare providers during the first two years following diagnosis, and approximately three quarters had no somatic hospitalizations in this period. In contrast, one third of the population experienced more than 30 psychiatric hospitalization days. Less than half had no somatic outpatient visits, whereas approximately 40% had more than 25 psychiatric outpatient visits.

**Table 1:** Characteristics at inclusion and during the first two years after diagnosis population

Total	*N* = 2,598	
	*n*	(%)
**Sex**		
Female	1,049	(40.4)
Male	1,548	(59.6)
**Age**		
18–21	1,001	(38.5)
22–25	748	(28.8)
26–35	849	(32.7)
**Civil status**		
Unmarried	2,174	(83.7)
Married/registered partnership	278	(10.7)
Divorced /widowed	146	(5.6)
**Employment status at baseline**		
Employed or enrolled in education	564	(21.7)
Not employed or enrolled in education	2,034	(78.3)
**Psychotic disorder**		
Schizophrenia	1,046	(40.3)
Other psychotic disorders	1,552	(59.7)
**Primary healthcare consultations** **(0–2 years)**		
0–20 consultations	1,463	(56.3)
21–50 consultations	848	(32.6)
> 50 consultations	287	(11.1)
**Somatic hospitalization days (0–2 years)**		
None	1,940	(74.7)
≥ 1 days	658	(25.3)
**Psychiatric hospitalization days (0–2 years)**		
None	1,101	(42.4)
1–30 days	675	(26.0)
> 30 days	822	(31.6)
**Somatic outpatient visits (0–2 years)**		
None	996	(38.3)
1–3	1,069	(41.2)
> 3	533	(20.5)
**Psychiatric outpatient visits (0–2 years)**		
0–3	807	(31.1)
4–24	789	(30.4)
25–49	461	(17.7)
≥ 50	541	(20.8)

### Attachment to employment or education at 3- and 5-year follow-up

At the 3-year follow-up, 505 individuals (19.6%) were employed or enrolled in education, while 539 individuals (21.6%) were employed or enrolled in education at the 5-year follow-up. Across both time points, 342 of the 2,493 individuals (13.7%) maintained attachment to employment or education, whereas 1,806 individuals (72.4%) were not attached at either time point.

At the 3-year follow-up, 89 individuals (3.5%) had been granted disability pension. By the 5-year follow-up, a total of 231 individuals (9.3%) had received disability pension.

### Sociodemographic variables

At the 3-year follow-up, women had statistically significantly lower odds of being attached to employment or education compared to men in the adjusted model (Table 2). At the 5-year follow-up, a similar trend was observed; however, no statistically significant differences were found between men and women in either the crude or adjusted model (Table 3). Older individuals (aged 22–25 and 26–35) had lower odds of attachment compared to younger individuals in the crude analyses at both time points, however, these associations were not statistically significant in the adjusted analyses (Tables 2 and 3).

At both time points, individuals who were married or in a registered partnership had statistically significantly higher odds of being employed or engaged in education compared to unmarried individuals, whereas no statistically significant differences were observed for divorced or widowed individuals.

Individuals who were employed or enrolled in education at baseline had substantially higher odds of attachment to employment or education at both 3- and 5-year follow-up compared to those not attached at baseline.

**Table 2:** Association between baseline characteristics and attachment to employment or education at 3-year post-diagnosis

	Employed or enrolled in education *n* = 2577 *n* (%)	Crude		Adjusted Model^a^	
		OR	CI	OR	CI
**Total**	505 (19.6)				
**Sex**					
Male	299 (19.5)^b^	Reference		Reference	
Female	206 (19.7)	1.01	0.83;1.24	0.73	0.57;0.94
**Age**					
18–21	222 (22.4)	Reference		Reference	
22–25	132 (17.8)	0.75	0.59;0.95	0.76	0.58;1.00
26–35	151 (17.9)	0.76	0.60;0.95	0.82	0.62;1.07
**Civil status**					
Unmarried	393 (18.4)	Reference		Reference	
Married/registered partnership	81 (29.1)	1.82	1.38;2.42	1.56	1.12;2.17
Divorced/widowed	28 (19.2)	1.05	0.69;1.61	1.27	0.79;2.06
**Employment status at baseline**					
Not employed or enrolled in education	221 (11.0)	Reference		Reference	
Employed or enrolled in education	284 (51.7)	8.36	6.73;10.39	7.95	6.30;10.03
**Psychotic disorder**					
Other psychotic disorders	308 (20.0)	Reference		Reference	
Schizophrenia	197 (19.0)	0.94	0.77;1.14	0.90	0.70;1.15
**Primary healthcare consultations** **(0–2 years)**					
0–20 consultations	263 (18.1)	Reference		Reference	
21–50 consultations	189 (22.5)	1.31	1.06;1.61	1.25	0.97;1.62
> 50 consultations	53 (18.7)	1.04	0.75;1.44	1.18	0.79;1.78
**Somatic hospitalization days**					
None	409 (21.2)	Reference		Reference	
≥ 1 days	96 (14.8)	0.64	0.50;0.82	0.78	0.57;1.05
**Psychiatric hospitalization days**					
None	260 (23.7)	Reference		Reference	
1–30 days	126 (18.9)	0.75	0.59;0.95	0.68	0.52;0.90
> 30 days	119 (14.6)	0.55	0.43;0.70	0.48	0.36;0.63
**Somatic outpatient visits**					
None	204 (20.6)	Reference		Reference	
1–3	202 (19.1)	0.91	0.73:1.13	1.14	0.89;1.47
> 3	99 (18.7)	0.89	0.68:1.16	1.17	0.82;1.65
**Psychiatric outpatient visits**					
0–3	83 (10.4)	Reference		Reference	
4–24	176 (22.5)	2.51	1.89;3.33	2.04	1.50;2.78
25–49	124 (27.1)	3.21	2.36;4.36	2.23	1.58;3.16
≥ 50	122 (22.7)	2.53	1.87;3.43	1.56	1.09;2.25

^a^Adjusted model adjusted for all variables

^b^Percentages represent the proportion of individuals attached to employment or education within each category

**Table 3:** Association between baseline characteristics and attachment to employment or education at 5 years post-diagnosis

	Employed or enrolled in education *n* = 2,493 *n* (%)	Crude		Adjusted model^a^	
		OR	CI	OR	CI
**Total**	539 (21.6)				
**Sex**					
Male	312 (21.1)^b^	Reference		Reference	
Female	227 (22.4)	1.08	0.89;1.31	0.80	0.63;1.02
**Age**					
18–21	237 (24.7)	Reference		Reference	
22–25	144 (20.1)	0.77	0.61;0.97	0.79	0.60;1.03
26–35	158 (19.4)	0.73	0.58;0.92	0.79	0.61;1.03
**Civil status**					
Unmarried	413 (20.0)	Reference		Reference	
Married/registered partnership	101 (36.6)	2.32	1.77;3.03	2.11	1.54;2.88
Divorced/widowed	25 (17.1)	0.83	0.53;1.29	0.92	0.56;1.51
**Employment status at baseline**					
Not employed or enrolled in education	257 (13.2)	Reference		Reference	
Employed or enrolled in education	282 (52.0)	7.15	5.77;8.85	6.19	4.94;7.77
**Psychotic disorder**					
Other psychotic disorders	327 (22.0)	Reference		Reference	
Schizophrenia	212 (21.1)	0.95	0.78;1.15	0.77	0.61;0.98
**Primary healthcare consultations** **(0–2 years)**					
0–20 consultations	282 (20.0)	Reference		Reference	
21–50 consultations	201 (24.8)	1.32	1.07;1.62	1.19	0.93;1.52
> 50 consultations	56 (20.4)	1.03	0.74;1.41	1.03	0.69;1.53
**Somatic hospitalization days**					
None	423 (22.7)	Reference		Reference	
≥1 days	116 (18.4)	0.77	0.61;0.97	1.00	0.75;1.33
**Psychiatric hospitalization days**					
None	268 (25.4)	Reference		Reference	
1–30 days	137 (21.3)	0.80	0.63;1.00	0.77	0.58;1.01
> 30 days	134 (16.8)	0.59	0.47;0.75	0.53	0.40;0.69
**Somatic outpatient visits**					
None	225 (23.6)	Reference		Reference	
1–3	208 (20.3)	0.82	0.67:1.02	0.98	0.77;1.25
> 3	106 (20.7)	0.84	0.65:1.10	0.96	0.69;1.35
**Psychiatric outpatient visits**					
0–3	77 (10.0)	Reference		Reference	
4–24	187 (24.7)	2.95	2.21;3.93	2.56	1.87;3.49
25–49	120 (26.9)	3.30	2.41;4.53	2.55	1.80;3.62
≥ 50	155 (29.6)	3.78	2.80;5.11	2.87	2.02;4.09

^a^Adjusted model adjusted for all variables

^b^Percentages represent the proportion of individuals attached to employment or education within each category

### Clinical variables

No statistically significant differences in attachment to employment or education were observed between individuals with schizophrenia and those with other psychotic disorders at the 3-year follow-up. However, in the adjusted model at the 5-year follow-up, schizophrenia was associated with statistically significantly lower odds of attachment to employment or education compared with other psychotic disorders (Tables 2 and 3).

In the adjusted analyses, no statistically significant association was observed between primary healthcare visits during the first two years and attachment at either follow-up, whereas higher odds were observed in the crude analysis among individuals with 21–50 visits. Also, there was no statistically significant association between somatic hospitalization days during the first two years post-diagnosis and attachment at 3- or 5-year follow-up in the adjusted analyses; however, in the crude analyses, somatic hospitalization days were associated with statistically significantly lower odds of attachment at both follow-up time points. In contrast, individuals with psychiatric hospitalization during the first two years, both 1–30 days and more than 30 days, had statistically significantly lower odds of attachment at the 3-year follow-up, while those with more than 30 days also had statistically significantly lower odds at the 5-year follow-up.

Somatic outpatient visits during the first two years post-diagnosis were not associated with attachment to employment or education at any of the two follow-up time points. In contrast, individuals who attended more than three psychiatric outpatient visits during the first two years post-diagnosis had statistically significantly higher odds of attachment to employment or education at both the 3- and 5-year follow-up compared with those who attended 0–3 visits.

## Discussion

This study investigated the association between clinical and sociodemographic factors and attachment to employment or the educational system among individuals with first-episode psychosis. Among individuals alive and eligible for follow-up, approximately 20% were employed or enrolled in education three years after diagnosis, increasing slightly to 22% at the 5-year follow-up. These estimates were based on follow-up-specific analytical populations derived from a population-based cohort of 2,633 individuals diagnosed in the Central Region of Denmark. These findings underscore the importance of identifying factors that contribute to the challenges individuals with psychotic disorders face in maintaining attachment to employment or education – an essential aspect of recovery.

Our results indicate that both clinical and sociodemographic factors influence attachment. Notably, being employed or enrolled in education at baseline strongly predicted future attachment. Individuals who were employed or enrolled in education at baseline had substantially higher odds of remaining engaged in education or employment at both three and five years post-diagnosis. However, it is important to keep in mind that several studies have found that the risk of declining employment both one and three years after first hospital admission for first-episode psychosis remains substantial, even among individuals who were employed at the time of hospitalization [31, 32], emphasizing the importance of continued support and intervention [33, 34].

Marital status was also a statistically significant factor in attachment to employment or education. Individuals who were married or in a registered partnership had twice the odds of being employed or enrolled in education at the 5-year follow-up. The protective effect was observed at the 3-year follow-up, though with slightly lower odds. These findings are consistent with previous research highlighting the role of strong social support in promoting recovery and functioning for individuals with psychosis [16, 35]. In crude analyses, older individuals had lower odds of attachment to employment or education compared with the youngest age group; however, these differences were attenuated after adjustment. One possible explanation is that younger individuals are more likely to be enrolled in education rather than employment, and educational settings may offer greater flexibility and support than the labor market for individuals experiencing mental illness, although the present study cannot directly distinguish between these mechanisms.

In the present study, no statistically significant differences in attachment to education or employment were observed between diagnostic groups at the 3-year follow-up; however, at 5 years, individuals with schizophrenia had statistically significantly lower odds of attachment than those with other psychotic disorders. These findings align with previous research indicating that schizophrenia is associated with higher risk of long-term disengagement from education or employment compared with other psychotic disorders [35]. The relatively attenuated differences observed between diagnostic groups, particularly at the earlier follow-up, contrast with findings from Finnish register-based research reporting substantially lower rates of long-term exclusion from employment, education, or training among individuals with non-schizophrenia psychoses compared with those with schizophrenia [35]. Several factors may contribute to this discrepancy. First, differences in study populations and outcome definitions may play a role, as the Finnish study included training as part of employment. Second, diagnostic classification in routine clinical practice may evolve over time, such that some individuals initially diagnosed with other psychotic disorders later receive a diagnosis of schizophrenia [36]. This diagnostic instability may have attenuated differences between diagnostic groups in the present study, particularly at earlier follow-up points.

Healthcare utilization patterns were differentially associated with attachment to education or employment. More frequent psychiatric outpatient contacts during the first two years following diagnosis were associated with higher odds of attachment at both the 3- and 5-year follow-up, whereas neither somatic outpatient visits nor primary healthcare contacts were independently associated with attachment after adjustment. This pattern suggests that sustained engagement with specialized psychiatric outpatient care may be particularly important for supporting functioning outcomes, potentially by facilitating symptom management, continuity of care, and early intervention in emerging difficulties. Evidence from randomized studies supports this interpretation, demonstrating that early intervention services in psychosis are associated with improved functioning, including greater participation in work or education and lower rates of hospitalization and treatment discontinuation compared with standard care [37]. From a mental healthcare system perspective, the observed variation in outpatient contacts warrant careful interpretation. Although national guidelines recommend timely and intensive specialized outpatient care following the first episode of psychosis, variation in the intensity and continuity of outpatient contacts was observed. This may reflect differences in service organization, transitions between specialized and standard care, and challenges related to continuity of care [38]. Importantly, variation in outpatient contact may also reflect differences in illness severity and symptom burden. Individuals with a more severe disease course may have limited capacity to attend appointments consistently, particularly during periods of clinical instability [39]. Thus, lower outpatient contact should not necessarily be interpreted as insufficient service provision alone but may also indicate greater vulnerability. Regardless of the underlying mechanisms, the results underscore the importance of ensuring accessible and flexible psychiatric outpatient service during the early course of psychosis, as this period appears critical for long-term attachment to employment and education [37].

In contrast, psychiatric hospitalization days during the first two years following diagnosis was associated with lower odds of attachment to employment or education at both the 3- and 5- year follow-up, particularly among individuals with longer hospital stays. Somatic hospitalizations were not independently associated with attachment after adjustment. These findings are consistent with previous research indicating that psychiatric hospitalizations, especially frequent or prolonged admissions, are associated with poor functioning outcomes, including disengagement from the labor market or educational system [14, 40]. Psychiatric hospitalizations may reflect a more severe illness trajectory, further highlighting the importance of early and continuous outpatient support during the recovery phase.

This study adds to the growing evidence demonstrating that sociodemographic factors, such as marital status and baseline employment or educational participation, as well as clinical factors related to illness severity and service engagement, are crucial for long-term attachment to employment or education among individuals with psychotic disorders. These findings highlight the importance of early interventions targeting vocational outcomes alongside sustained and adequately resourced psychiatric outpatient care to support long-term functioning outcomes [10].

When interpreting the results of this population-based cohort study strengths and limitations must be considered. Among the key strengths is the use of population-based Danish registries, enabling comprehensive registry-based follow-up of the study cohort. The large cohort and reliance on national registries minimize the risk of recall and selection bias and provide a robust foundation for examining long-term outcomes. To capture a more comprehensive picture of functioning, outcomes were assessed at both three and five years post-diagnosis. This approach was chosen because individuals diagnosed with a psychotic disorder may follow diverse illness trajectories and may experience severe and disabling symptoms that limit their ability to participate in employment or education.

A potential limitation lies in the categorization of individuals into only two diagnostic groups: schizophrenia and other psychotic disorders. The latter represents a heterogeneous group, within which the course and severity of illness can vary considerably depending on the specific diagnosis. Findings related to this group should therefore be interpreted with caution. Furthermore, information on comorbid substance use disorders was not included, although this may influence functioning outcomes. Analyses were restricted to individuals alive at each follow-up, which may have led to an overestimation of the proportion of individuals attached to employment or education, as individuals who died during follow-up are likely to have had a more severe illness trajectories and disability. Although relatively few individuals died during follow-up, the results should still be interpreted with caution. In addition, some individuals were registered as emigrated during follow-up. In Danish administrative data, emigration status may reflect loss of a registered address rather than confirmed residence abroad and is therefore likely to capture a subgroup with limited attachment to employment or education rather than representing true loss to follow-up.

This study was conducted using available data on individuals living in Denmark, where a comprehensive social welfare system provides financial support through benefits such as state educational grants, unemployment benefits, sickness benefits, and disability pensions. These benefits aim to provide financial support during periods of unemployment, illness, education, or disability, ensuring a basic standard of living for all citizens. Furthermore, the public welfare system provides support through both work-related and social programs, which may contribute to greater stability in everyday life for individuals in need. This contextual factor should be considered when interpreting the results, as welfare support may function both as a facilitator of labor market or educational attachment and as a potential disincentive, given that income security can be maintained outside employment or education. Consequently, patterns of work and educational affiliation may differ in countries where comparable welfare systems are not available.

In conclusion, this population-based cohort study found that sociodemographic factors, such as marital status and employment or educational participation at diagnosis, together with sustained engagement in psychiatric outpatient care, were associated with higher odds of participation in education or the labor market both at three and five years after diagnosis. These findings highlight the importance of early intervention and continued access to adequately resourced psychiatric outpatient services to enhance long-term functioning recovery among individuals with psychotic disorders.

## Data Availability

Data are not available due to legal restrictions, as the data are owned by CROSS-TRACKS.
